# Inadequate Physical Activity Is Associated with Worse Physical Function in a Sample of COVID-19 Survivors with Post-Acute Symptoms

**DOI:** 10.3390/jcm12072517

**Published:** 2023-03-27

**Authors:** Vincenzo Galluzzo, Maria Beatrice Zazzara, Francesca Ciciarello, Matteo Tosato, Anna Maria Martone, Cristina Pais, Giulia Savera, Riccardo Calvani, Anna Picca, Emanuele Marzetti, Francesco Landi, on behalf of Gemelli Against COVID-19 Post-Acute Care Team

**Affiliations:** 1 Fondazione Policlinico Universitario “Agostino Gemelli” IRCCS, 00168 Rome, Italy; 2 Department of Geriatrics and Orthopedics, Università Cattolica del Sacro Cuore, 00168 Rome, Italy

**Keywords:** six-minute walking test, one-minute sit-to-stand test, muscle strength, Long COVID, geriatrics

## Abstract

**Background**—Patients affected by Long COVID often report shorter times spent doing physical activity (PA) after COVID-19. The aim of the present study was to evaluate potential associations between PA levels and parameters of physical function in a cohort of COVID-19 survivors with post-acute symptoms, with a particular focus on individuals aged 65 and older. **Materials and methods**—PA levels before and after COVID-19 were assessed in a sample of patients that had recovered from COVID-19 and were admitted to a post-acute outpatient service at the Fondazione Policlinico Universitario Agostino Gemelli IRCCS (Rome, Italy). Participation in PA was operationalized as the engagement in leisure-time PA for at least 150 min per week in the last 3 months. Self-rated health and measures of physical performance and muscle strength were assessed. **Results**—Mean age of 1846 participants was 55.2 ± 14.4 years and 47% were women. Before COVID-19, inactivity was detected in 47% of the whole study population; only 28% maintained pre-COVID-19 PA engagement. Inactivity was more frequent in women. The stopping of physical activity was associated with increased BMI and CRP levels, lower vitamin D levels and a higher prevalence of post-COVID-19 fatigue, dyspnea, arthralgia, and myalgia. Active participants had higher handgrip strength and performed better on both the six-minute walking test (6MWT) and at the one-minute sit-to-stand test (1MSTST). In particular, at the 6MWT, participants 65 and older that were still active after COVID-19 walked 32 m more than sedentary peers. Moreover, the distance covered was 28 m more than those who were active only before COVID-19 (*p* = 0.05). Formerly active subjects performed similarly at the 6MWT to inactive participants. PA was associated with better self-rated health. **Conclusions**—Our findings reveal that inactivity is frequent in the post-acute COVID-19 phase. Stopping physical activity after COVID-19 results in measures of performance that are comparable to those who were never active. Relevant differences in the distance covered at the 6MWT were found between older active subjects and their sedentary peers.

## 1. Introduction

Physical activity (PA) represents one of the major determinants of health worldwide [1]. Starting regular aerobic PA reduces the incidence of several chronic conditions such as cardiovascular disease (CVD), type 2 diabetes (T2DM), depression, and dementia [2,3]. Regardless of its intensity, any type of PA that reduces sedentary time may decrease the risk of premature mortality of young and older adults [4]. Even a reduction of 10% of inactivity can prevent more than 500,000 deaths every year [1]. Moreover, following an acute injury, one of the strategies to facilitate the process of recovery is based on muscle training [5,6,7].

PA plays a role in modulating the immune system, and the exercise-induced anti-inflammatory response seems to be one of the mechanisms implicated in the protective role of PA against several conditions, including infectious diseases [8]. A recent meta-analysis has shown that regular PA is associated with a one-third reduction in the risk of community-acquired infectious diseases [9]. Furthermore, the rate of upper respiratory tract infections tends to be lower in subjects who perform aerobic exercises for a greater number of days per week [10]. Concerning viral infections, it seems that regular PA may decrease the risk of influenza-associated mortality [11]. 

During the COVID-19 pandemic, measures taken by governments to control the SARS-CoV-2 transmission have changed the lifestyle habits of millions of people worldwide [12]. If compared to the pre-pandemic era, people experienced a reduction in PA levels as a result of lockdown restrictions [13,14,15]. Sedentary habits are associated with a higher risk of hospitalization, admission to the intensive care unit (ICU), and mortality due to COVID-19 [16]. On the other hand, regular PA may decrease the risk of SARS-CoV-2 infection and severe COVID-19 symptoms [3,17]. Even in the acute phase, moderate-intensity aerobic exercises might reduce the severity and progression of COVID-19 [18].

Little is known about the role of PA in the post-acute phase of COVID-19. Patients affected by Long COVID, which is characterized by long-lasting symptoms [19], often report a shorter time spent doing PA compared to pre-COVID-19 [20]. Reduced walking and exercise tolerance are among the most common symptoms in those that have been hospitalized for COVID-19 [21]. After being discharged from the hospital, COVID-19 survivors appear to have worse physical performance, particularly in the first few months after the infection [22].

The aim of the present study was to examine the prevalence of physical activity levels before and after the viral infection in a cohort of hospitalized or home-managed COVID-19 survivors with post-acute symptoms, and its association with measures of physical performance, muscle strength, and self-rated health. 

## 2. Materials and Methods 

The Gemelli Against COVID-19 Post-Acute Care (GAC19-PAC) project is an ongoing initiative developed by the Department of Geriatrics, Neuroscience, and Orthopedics of the Catholic University of the Sacred Heart (Rome, Italy) to analyze long-term sequalae of COVID-19 and their impact on overall health, quality of life, and physical/cognitive performance. The post-acute outpatient service, called “Day Hospital post-COVID-19”, has been established in April 2020 at the Fondazione Policlinico Universitario Agostino Gemelli IRCCS (Rome, Italy) for people who have recovered from COVID-19 [23].

### 2.1. Study Sample

The study population included individuals aged > 18 years old that have officially recovered from COVID-19 and have been admitted to the post-COVID-19 outpatient service between 21 April 2020 and 31 December 2021. At the moment of the evaluation, all patients met the World Health Organization (WHO) criteria for discontinuation of quarantine [24].

### 2.2. Data Collection

Patients were offered a comprehensive medical assessment. Clinical parameters, anthropometric measures, medical history, current medications, and lifestyle habits including physical activity, were collected in a structured electronic database. Long-term symptoms of SARS-CoV-2 infection were evaluated with a multidisciplinary approach [25,26,27,28,29].

Body weight was measured through an analogue medical scale. Body height was measured using a standard stadiometer. Body mass index (BMI) was defined as weight (kilograms) divided by the square of height (meters). 

COVID-19 severity was considered as follows: (1) no hospitalization; (2) hospitalization with no oxygen supplementation; (3) hospitalization with low-flow oxygen supplementation; (4) hospitalization with noninvasive ventilation (NIV) or ICU admission with invasive ventilation.

Symptoms that arose during or immediately after the SARS-CoV-2 infection and persisted at the time of the evaluation have been included among sequalae of acute COVID-19. 

### 2.3. Physical Activity Levels

All participants were interviewed concerning their usual physical activity levels prior to acute SARS-CoV-2 infection and at the time of the evaluation. Regular participation in physical activity was operationalized as the engagement in aerobic PA, associated or not with resistance training, for a minimum of 150 min per week in the last 3 months [30]. Consequently, the subjects were divided into three groups: (1) inactive, not physically active before COVID-19 and at the time of the evaluation; (2) formerly active, subjects who practiced regular PA only before COVID-19 and not at the time of the evaluation; (3) active, subjects who practiced regular PA both before and after COVID-19.

### 2.4. Hematological Parameters

Vitamin D, plasma albumin, C-reactive protein (CRP), and hemoglobin levels were detected using standard biochemistry methods on fully automated testing systems.

### 2.5. Assessment of Muscle Strength and Physical Performance 

Upper extremity muscle strength was measured by handgrip strength testing using a North Coast hand-held hydraulic dynamometer (North Coast Medical, Inc., Morgan Hill, CA, USA) [31]. After one familiarization trial, muscle strength was measured in both hands and the higher value (kg) was used for the analysis [31]. 

Physical performance was evaluated by the one-minute sit-to-stand test (1MSTST) and the six-minute walking test (6MWT). These tests are commonly used to assess exercise-induced respiratory impairment [32]. For the 1MSTST, participants must stand up from a chair and sit down for 60 s as quickly as possible. A higher number of rises at the 1MSTST and a longer distance covered at the 6MWT (m) indicate better performance [33,34].

### 2.6. Self-Rated Health

A visual analog scale (VAS) was used to rapidly assess self-rated health on a scale from 0 to 100, with 0 corresponding to the worst perception of health status and 100 indicating the best imaginable health [35].

### 2.7. Statistical Analyses

Continuous variables were expressed as mean ± standard deviation (SD), and categorical variables were expressed as frequencies by absolute value and percentage (%) of the total. Descriptive statistics were used to describe clinical characteristics of the study population according to physical activity levels. Differences in the proportions and means of covariates between study participants across different physical activity categories were assessed using Fisher’s Exact Test and *t* test statistics (*p* for trend), respectively. 

Analysis of covariance (ANCOVA) was used to examine the association between physical activity levels and measures of physical performance, muscle strength, and self-rated health, after adjusting for potential confounding variables. Variables considered as covariates were chosen according to their clinical significance and/or their significant difference at the univariate analyses between inactive, formerly active, and active participants. The interactions of covariates on the relationship between physical function’s measures and physical activity levels were tested by adding the interaction term into the adjusted models. No significant interactions were reported.

All analyses were performed using SPSS software (version 11.0, SPSS Inc., Chicago, IL, USA). 

## 3. Results

Between April 2020 and December 2021, 1921 individuals were admitted to the post-COVID-19 outpatient service. For the present study, 75 subjects were excluded for missing values in the variables of interest; thus, a sample of 1846 subjects was considered. The mean age of the study sample was 55.2 ± 14.4 years (range 18–86) and 47% were women. Characteristics of the study population according to physical activity levels are summarized in Table 1.

**Figure 1:**
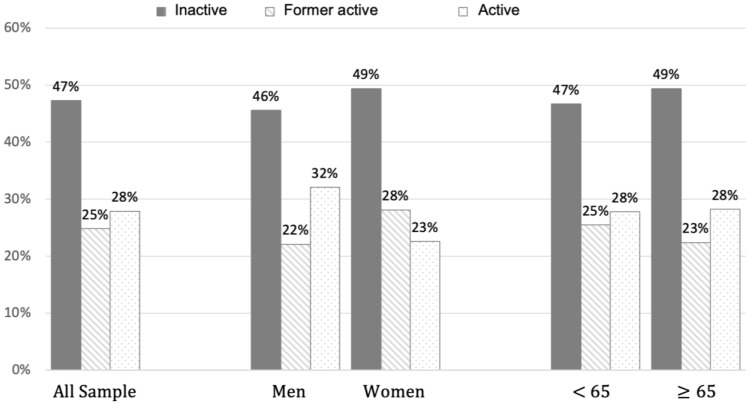
Prevalence of PA levels according to sex and age groups.

Inactive people were 47% of the whole study population; 53% were physically active before COVID-19, but only 28% maintained usual physical activity levels at the moment of the evaluation (Figure 1). In particular, only 23% of females continued to exercise after COVID-19, while the prevalence in the male subgroup was about 32%. The proportion of participants who practiced PA at the time of the evaluation was similar across ages (Figure 1).

The average number of days from COVID-19 diagnosis to admission to the outpatient service were similar between inactive and active subjects (85.2 ± 42.7 versus 91.3 ± 34.8, respectively; *p* = 0.1). COVID-19 severity had the same distribution in the three groups of interest (inactive, formerly active, and active), except for those who needed invasive ventilation, in which the percentage of inactive subjects was higher (*p* = 0.01). Compared to active participants, subjects in the inactive and formerly active groups were older and had a higher prevalence of hypertension, diabetes, and chronic obstructive pulmonary disease (COPD). The highest prevalence of unemployed individuals was found in the active group, while retired people were more common in the other two groups. Active and formerly active groups presented the same percentage of people occupied as housewives and declared a similar number of kids. Post-acute COVID-19 fatigue and dyspnea were less common amongst active individuals. Persistent arthralgia and myalgia were more common in formerly active participants. Formerly active and active subjects, especially those who maintained usual levels of PA after COVID-19, showed lower BMI and C-reactive protein (CRP) levels, and higher vitamin D levels. 

Unadjusted and adjusted ANCOVA models were performed to evaluate the association of physical activity levels with measures of physical performance, muscle strength, and self-rated health (Table 2 and Table 3). In the whole sample, practicing PA at the moment of the evaluation was associated with a longer distance covered during the 6MWT, greater numbers of repetitions at the 1MSTST, and higher values at the handgrip strength. Active participants 65 and older walked, on average, 28 m more than formerly active groups and 32 m more than sedentary peers (457.1 m vs. 428.8 m vs. 425.0 m, respectively; *p* = 0.05). Sedentary habits of inactive and formerly active individuals were associated with a similar distance covered at the 6MWT. Older people who were still active reported better scores of self-rated health compared to all inactive subjects.

## 4. Discussion

In the present study, we investigated the prevalence of PA in post-acute COVID-19 patients and its potential association with functional parameters. The proportion of inactive subjects in our cohort of COVID-19 survivors with persistent symptoms was higher compared to the healthy Italian population before the pandemic (72% vs. 36.8%, respectively) [36]. Following lockdown restrictions, even those who did not test positive to SARS-CoV-2 swab experienced a strong reduction in PA levels [36]. In our study, participants who stopped practicing PA represented almost 25% of the sample. These findings are consistent with those shown by a recent Dutch-Belgian survey, in which people who had not returned to their usual PA after COVID-19 were approximately 24.7% [20]. 

We found that inactivity after COVID-19 was more frequent amongst women, although these results were not explained with major engagement in job and/or home responsibilities (numbers of kids, work as a housewife). Most likely, women experience post-acute COVID-19 symptoms more than men [21,37]. Frequently reported Long COVID-19 symptoms are fatigue, dyspnea, and joint-muscle pain [38,39,40], which  are associated with a reduced tolerance to exercise [41,42,43] that may limit physical activity [21,38,44]. 

We found no substantial differences in the prevalence of inactivity after COVID-19 according to age. *Lombardo* et al. have shown that the incidence of post-COVID-19 fatigue, dyspnea, and joint-muscle pain is lower at young ages (<47 years old), but it tends to be similar among adults (47–58 years old) and older adults (>58) [45].

Our findings revealed a positive trend in BMI and CRP levels from sedentary to active subjects. It is noteworthy that—as a consequence of unhealthy dietary habits and reduction in PA—many adults gained weight during lockdown restrictions [46]. Moreover, evidences have demonstrated an association between PA and inflammatory mediators’ levels [47,48] that may be attributed to the reduction of visceral fat mass [49,50], showing that PA is involved in maintaining the balance between pro-inflammatory and anti-inflammatory responses [51].

Vitamin D levels were higher among physically active participants, particularly in those who did not stop practicing physical activity after COVID-19. Consistent with our findings, a correlation between normal vitamin D levels and habitually practicing PA has been identified in a cohort of 86 forensic inpatients [52]. However, evidence is contradictory with other studies showing no relationships [53,54,55,56]. Similar contrasting results involve the relationship between vitamin D levels and Long COVID symptoms [29,57], thus further research is warranted. 

In the present investigation, we found that PA was associated with better physical performance and higher values of muscle strength. Differences in the distance covered during the 6MWT were found particularly in the older subgroup, in which active participants walked 32 m more than inactive peers and 28 m more than formerly active subjects. According to Bohannon and Crouch, a minimum change of 30.5 m in the distance covered at the 6MWT may be considered clinically meaningful [58]. These results are in line with several studies demonstrating that both aerobic and resistance training might improve physical function, protecting the body from physical frailty and maintaining independence later in life [59]. Moreover, we found that individuals who stopped practicing PA after COVID-19 walked a similar distance in the 6MWT to sedentary subjects, and more frequently manifested post-acute COVID-19 symptoms compared to active subjects. Paneroni et al. have demonstrated that patients with any previous disabilities but recent hospitalization for COVID-19 pneumonia, exhibit lower values of bicep strength and smaller numbers of rises at the 1MSTST (15 kg and 22.1, respectively) [60]. Notably, sarcopenia can be detected in a relevant proportion of people who have recovered from the SARS-CoV-2 infection [61]. Due to the complexity of COVID-19 and its implications, we cannot rule out that a combination of both long-term symptoms and less involvement in PA may influence muscle function in the first months after the infection [21,22,61]. 

In our sample, physically active participants declared better self-rated health. Older subjects who maintained usual PA levels after COVID-19 manifested better scores on their quality of life than sedentary participants. Several RCTs found that changing the PA regimen may reduce psychological distress and improve the quality of life among community-dwelling older adults [62,63]. Considering that government restrictions during the pandemic have drastically worsened the lifestyle habits and mobility of older people [64], practicing PA or recovering usual PA levels may represent a solution to ameliorate psychological well being.

Maintaining usual PA levels after the infection is crucial. Lockdown restrictions and the fear of contagion have influenced choices about the well being of millions of people worldwide. The large cohort of Long COVID patients enrolled, and the attempt to emphasize the potential role of PA and persistent symptoms, including fatigue, in determining muscle function in the post-acute phase of the infection represent the strengths of this study.

Some methodological issues should be taken into account in the interpretation of the results. A major limitation of cross-sectional studies is the lack of longitudinal data that capture changes on an individual level over time, thus not allowing a thorough follow-up of post-COVID-19 symptoms and PA levels. The observational nature of our study design prevents the evaluation of cause-effect relationship between sedentary habits and poor physical function in patients with Long COVID. Therefore, we cannot assert whether persistent symptoms influence physical performance more than PA levels.

Furthermore, information about the type, volume, and intensity of activity practiced before COVID-19 and/or maintained after COVID-19 were not assessed. Data on dietary habits and physical function before COVID-19 that may have influenced patients’ health status and physical function after the infection were not available. Moreover, our sample included predominantly symptomatic patients attending a post-COVID-19 outpatient service, which may induce a selection bias. Indeed, we cannot determine if further factors might be implicated in the deterioration of physical function after COVID-19. The involvement of different anatomical districts that follows the SARS-CoV-2 infection shows us the complexity of this illness and the difficulty in understanding the pathogenesis of long-term sequalae. Finally, the long period of enrollment does not allow us to distinguish whether specific SARS-CoV-2 variants might have influenced post-COVID health status more than others, although following the spread of variants in Italy during this period of time, our sample is unlikely to contain individuals infected with the omicron variant, which is likely the variant associated with a fewer number of persistent symptoms [65].

In conclusion, we analyzed the prevalence of physical activity levels and its association with parameters of physical function in a sample of COVID-19 survivors with post-acute symptoms. We found a high prevalence of inactivity after COVID-19, especially in women. Lower BMI and CRP levels, and higher vitamin D levels were more common in active people. Post-acute COVID-19 symptoms were less frequent in active people. Being physically active, particularly after COVID-19, was associated with better performance on the 6MWT, a higher number of repetitions at the 1MSTST, and higher values of handgrip muscle strength. Differences in the distance covered at the 6MWT were relevant between active and inactive older people. We found that formerly active subjects performed similarly to habitually sedentary subjects. Moreover, physical activity was associated with higher values of self-rated health.  Future studies are warranted to further assess associations between physical activity levels and muscle function in the post-acute COVID-19 and to evaluate whether improving exercise training may ameliorate functional outcomes after the SARS-CoV-2 infection.

## Figures and Tables

**Table 1:** Characteristics of study population according to physical activity level *.

	Physical Activity Level				
	Total Sample (*n* = 1846)	Inactive (*n* = 873)	Formerly Active (*n* = 458)	Active (*n* = 515)	*p* for Trend
***General and clinical characteristics***					
**Age** (years)	55.2 ± 14.4	56.3 ± 14.4	53.6 ± 14.6	54.6 ± 14.0	0.02
**Gender**					<0.001
Male	987 (53)	449 (51)	217 (47)	321 (62)	
Female	859 (47)	424 (49)	241 (53)	194 (38)	
**Education** (years)	13.9 ± 4.4	13.6 ± 5.2	12.5 ± 3.4	14.8 ± 3.8	0.65
**Marital status**					0.02
Married	1095 (59)	539 (62)	247 (54)	309 (60)	
In other type of relationship	104 (6)	51 (6)	32 (7)	21 (4)	
Divorced/Separated	239 (13)	103 (11.5)	64 (14)	72 (14)	
Widowed	119 (6)	60 (7)	28 (6)	31 (6)	
Single	289 (16)	120 (13.5)	87 (19)	82 (16)	
**Cohabitation status**					0.01
Alone	256 (13.5)	96 (11)	78 (17)	82 (16)	
With partner and sons	788 (42.5)	384 (44)	192 (42)	212 (41)	
With only the partner	517 (28)	244 (28)	124 (27)	149 (29)	
With only the sons	142 (8)	79 (9)	27 (6)	36 (7)	
With parents	93 (5)	35 (4)	32 (7)	26 (5)	
With brothers	9 (0.5)	9 (1)	0	0	
With other relatives	14 (1)	9 (1)	0	5 (1)	
With others (not relatives)	27 (1.5)	17 (2)	5 (1)	5 (1)	
**Number of kids**	1.55 ± 1.06	1.63 ± 1.04	1.48 ± 1.08	1.47 ± 1.10	0.05
**Occupational status**					<0.001
Employed	1263 (68)	585 (67)	312 (68)	366 (71)	
Unemployed	198 (11)	83 (9.5)	41 (9)	74 (14)	
Retired	336 (18)	183 (21)	87 (19)	66 (13)	
Other	49 (3)	22 (2.5)	18 (4)	9 (2)	
Housewife	99 (5.5)	65 (7.5)	16 (3.5)	18 (3.5)	
**Hypertension**	612 (33)	332 (38)	142 (23)	138 (23)	<0.001
**Heart failure**	39 (2)	28 (3)	6 (1)	5 (1)	0.008
**Diabetes**	180 (10)	104 (12)	34 (7)	42 (8)	0.01
**Renal failure**	42 (2)	28 (3)	9 (2)	5 (1)	0.02
**COPD**	126 (7)	86 (10)	15 (4)	24 (5)	<0.001
**Cancer**	29 (2)	13 (2)	6 (1)	10 (2)	0.70
**BMI** (kg/m^2^)	26.5 ± 4.8	27.5 ± 5.2	25.9 ± 4.5	25.5 ± 3.9	<0.001
*Severity of COVID-19 during acute phase*					
**Home**	786 (43)	347 (40)	224 (49)	215 (42)	0.01
**Hospital—no O_2_ support**	203 (11)	97 (11)	49 (11)	57 (11)	
**Hospital—O_2_ support**	394 (21)	179 (21)	90 (20)	125 (24)	
**Hospital—NIV, CPAP**	355 (19)	186 (21)	75 (16)	94 (19)	
**Hospital—Invasive Ventilation**	108 (6)	64 (7)	20 (4)	24 (4)	
**Length of hospital stay**	19.2 ± 12.3	19.8 ± 12.3	18.4 ± 12.4	18.7 ± 12.3	0.30
*Persistent symptoms after COVID-19*					
**Fatigue**	1164 (63)	567 (65)	298 (65)	299 (58)	<0.001
**Arthralgia**	645 (35)	297 (34)	188 (41)	160 (31)	<0.001
**Myalgia**	649 (35)	306 (35)	188 (41)	155 (30)	<0.001
**Dyspnea**	1205 (65)	585 (67)	321 (70)	299 (58)	<0.001
*Hematological parameters*					
**Albumin**	42.6 ± 3.0	42.2 ± 3.1	42.8 ± 3.1	43.0 ± 2.8	<0.001
**Hemoglobin**	14.1 ± 1.4	14.1 ± 1.5	14.0 ± 1.4	14.3 ± 1.4	<0.01
**Vitamin D**	24.6 ± 11.7	22.5 ± 11.1	25.7 ± 12.0	27.1 ± 11.9	<0.001
**C-reactive protein**	2.9 ± 6.0	3.5 ± 6.6	2.8 ± 5.7	2.1 ± 5.3	<0.001

* Data are given as number (percent) for gender, education, diseases, place, oxygen support, invasive ventilation; for all the other variables, means ± SD are reported. BMI: Body mass index. NIV: Non-Invasive Ventilation. CPAP: Continuous Positive Airways Pressure.

**Table 2:** Unadjusted and adjusted * means (SE) of physical performance, muscle strength measures, and self-rated health in men and women according to physical activity levels.

	Unadjusted			Adjusted *		
	Inactive (*n* = 873)	Formerly Active (*n* = 458)	Active (*n* = 515)	Inactive (*n* = 873)	Formerly Active (*n* = 458)	Active (*n* = 515)
**Physical performance measures**						
*Male*						
Six-minute walking test (m)	516.8 (6.48)	543.2 (7.51)	555.4 (7.14) ^#^	532.5 (5.61)	534.3 (7.47)	549.7 (6.52) ^
Chair stand test	25.1 (0.41)	25.4 (0.65)	27.6 (0.54) ^#^	25.6 (0.42)	25.3 (0.59)	27.2 (0.49) ^§^
*Female*						
Six-minute walking test (m)	490.3 (7.22)	519.5 (6.33)	526.4 (7.91) ^#^	506.5 (5.40)	511.9 (6.19)	524.3 (7.95) ^
Chair stand test	23.4 (0.46)	25.3 (0.58)	26.1 (0.79) ^#^	24.0 (0.51)	24.7 (0.64)	25.8 (0.70) ^
**Muscle strength measure**						
*Male*						
Handgrip Strength (kg)	35.1 (0.55)	35.6 (0.71)	37.5 (0.70) ^§^	35.6 (0.55)	35.7 (0.75)	37.4 (0.64) ^
*Female*						
Handgrip Strength (kg)	20.6 (0.34)	21.1 (0.41)	23.5 (0.59) ^#^	20.9 (0.39)	21.7 (0.46)	23.0 (0.54) ^#^
**Self-rated health**						
*Male*						
Visual Analogue Scale	82.7 (0.58)	85.3 (0.79)	87.4 (0.59) ^#^	83.0 (0.59)	84.9 (0.81)	86.5 (0.71) ^#^
*Female*						
Visual Analogue Scale	79.6 (0.76)	84.0 (0.83)	84.5 (0.49) ^#^	80.1 (0.78)	83.5 (0.98)	84.1 (1.12) ^#^

* Adjusted for age, hypertension, heart failure, COPD, diabetes, renal failure, BMI, albumin, C-reactive protein, vitamin D, hemoglobin, and acute COVID-19 severity. ^#^ *p* for trend <0.001; ^§^ *p* for trend = 0.01; ^ *p* for trend = 0.05.

**Table 3:** Unadjusted and adjusted * means of physical performance, muscle strength measures, and self-rated health in participants younger than 65 years or 65 years and older according to physical activity levels.

	Unadjusted			Adjusted *		
	Inactive (*n* = 873)	Formerly Active (*n* = 458)	Active (*n* = 515)	Inactive (*n* = 873)	Formerly Active (*n* = 458)	Active (*n* = 515)
**Physical performance measures**						
<*65 years*						
Six-minute walking test (m)	529.9 (4.19)	549.1 (4.28)	563.1 (5.59) ^#^	541.7 (3.90)	542.8 (4.77)	560.2 (5.02) ^#^
Chair stand test	25.5 (0.33)	26.4 (0.45)	28.1 (0.51) ^#^	26.0 (0.36)	26.1 (0.48)	27.6 (0.45) ^#^
≥*65 years*						
Six-minute walking test (m)	394.3 (12.9)	443.7 (13.2)	479.7 (11.1) ^#^	425.0 (12.1)	428.8 (16.1)	457.1 (15.4) ^
Chair stand test	19.7 (0.66)	21.1 (1.06)	23.1 (0.84) ^§^	20.3 (0.76)	21.4 (1.07)	23.1 (0.92) ^
**Muscle strength measure**						
<*65 years*						
Handgrip Strength (kg)	29.1 (0.49)	28.6 (0.64)	33.8 (0.69) ^#^	29.4 (0.49)	29.2 (0.64)	33.3 (0.63) ^#^
≥*65 years*						
Handgrip Strength (kg)	23.8 (0.72)	25.6 (0.87)	26.6 (0.85) ^	24.9 (0.72)	25.2 (0.95)	25.4 (0.88) °
**Self-rated health**						
<*65 years*						
Visual Analogue Scale	82.1 (0.54)	85.6 (0.62)	87.1 (0.56) ^#^	82.5 (0.54)	85.2 (0.70)	85.9 (0.70) ^#^
≥*65 years*						
Visual Analogue Scale	78.5 (0.98)	81.2 (1.36)	83.5 (1.07) ^#^	78.4 (1.07)	81.2 (1.47)	84.1 (1.38) ^#^

* Adjusted for age, hypertension, heart failure, COPD, diabetes, renal failure, BMI, albumin, C-reactive protein, vitamin D, hemoglobin, and acute COVID-19 severity. ^#^ *p* for trend <0.001; ^§^ *p* for trend = 0.01; ^ *p* for trend = 0.05; ° *p* for trend = ns.

## Data Availability

All the data and material are available.

## Appendix A

**The Gemelli Against COVID-19 Post-Acute Care Team**

Steering committee: Landi Francesco, Gremese Elisa

Coordination: Bernabei Roberto, Fantoni Massimo, Gasbarrini Antonio

*Field investigators*:

- Gastroenterology team: Porcari Serena, Settanni Carlo Romano
- Geriatric team: Benvenuto Francesca, Bramato Giulia, Brandi Vincenzo, Carfì Angelo, Ciciarello Francesca, Fabrizi Sofia, Galluzzo Vincenzo, Lo Monaco Maria Rita, Martone Anna Maria, Marzetti Emanuele, Napolitano Carmen, Pagano Francesco Cosimo, Pais Cristina, Rocchi Sara, Rota Elisabetta, Salerno Andrea, Tosato Matteo, Tritto Marcello, Zazzara Maria Beatrice, Calvani Riccardo, Catalano Lucio, Picca Anna, Savera Giulia, Damiano Francesco Paolo, Rocconi Alessandra, Galliani Alessandro, Spaziani Giovanni, Tupputi Salvatore, Cocchi Camilla, Pirone Flavia, D’Ignazio Federica, Cacciatore Stefano, Recupero Carla, Massaro Claudia, Elmi Daniele, Simoni Sofia, Ambrosio Fiorella, Gava Giordana, Tagliacozzi Elena, Ragozzino Rosa, Notari Maria Vittoria, Labriola Rosangela, Bulla Modestina, Giordano Giulia, Agostino Clara
- Infectious disease team: Cauda Roberto, Tamburrini Enrica, Borghetti A, Di Giambenedetto Simona, Murri Rita, Cingolani Antonella, Ventura Giulio, Taddei E, Moschese D, Ciccullo A, Dusina A, Seguiti C
- Internal Medicine team: Stella Leonardo, Addolorato Giovanni, Franceschi Francesco, Mingrone Gertrude, Zocco MA
- Microbiology team: Sanguinetti Maurizio, Cattani Paola, Marchetti Simona, Posteraro Brunella, Sali M
- Neurology team: Bizzarro Alessandra, Lauria Alessandra
- Ophthalmology team: Rizzo Stanislao, Savastano Maria Cristina, Gambini G, Cozzupoli GM, Culiersi C
- Otolaryngology team: Passali Giulio Cesare, Paludetti Gaetano, Galli Jacopo, Crudo F, Di Cintio G, Longobardi Y, Tricarico L, Santantonio M
- Pediatric team: Buonsenso Danilo, Valentini P, Pata D, Sinatti D, De Rose C
- Pneumology team: Richeldi Luca, Lombardi Francesco, Calabrese A, Leone Paolo Maria, Calvello Maria Rosaria, Intini Enrica, Montemurro Giuliano
- Psychiatric team: Sani Gabriele, Janiri Delfina, Simonetti Alessio, Giuseppin G, Molinaro M, Modica M
- Radiology team: Natale Luigi, Larici Anna Rita, Marano Riccardo
- Rheumatology team: Paglionico Annamaria, Petricca Luca, Varriano V, Gigante Luca, Natalello G, Fedele AL, Lizzio MM, Tolusso B, Di Mario Clara, Alivernini S
- Vascular team: Santoliquido Angelo, Santoro Luca, Di Giorgio Angela, Nesci Antonio, Popolla V

## References

[1] Lee I.M., Shiroma E.J., Lobelo F., Puska P., Blair S.N., Katzmarzyk P.T., Lancet Physical Activity Series Working Group (2012). Effect of physical inactivity on major non-communicable diseases worldwide: An analysis of burden of disease and life expectancy. Lancet.

[2] Wahid A., Manek N., Nichols M., Kelly P., Foster C., Webster P., Kaur A., Smith C.F., Wilkins E., Rayner M. (2016). Quantifying the Association Between Physical Activity and Cardiovascular Disease and Diabetes: A Systematic Review and Meta-Analysis. J. Am. Heart Assoc..

[3] National Center for Chronic Disease Prevention and Health Promotion (NCCDPHP) (2020). Physical Activity Prevents Chronic Disease.

[4] Ekelund U., Tarp J., Steene-Johannessen J., Hansen B.H., Jefferis B., Fagerland M.W., Whincup P., Diaz K.M., Hooker S.P., Chernofsky A. (2019). Dose-response associations between accelerometry measured physical activity and sedentary time and all cause mortality: Systematic review and harmonised meta-analysis. BMJ.

[5] Bayer M.L., Hoegberget-Kalisz M., Jensen M.H., Olesen J.L., Svensson R.B., Couppé C., Boesen M., Nybing J.D., Kurt E.Y., Magnusson S.P. (2018). Role of tissue perfusion, muscle strength recovery, and pain in rehabilitation after acute muscle strain injury: A randomized controlled trial comparing early and delayed rehabilitation. Scand. J. Med. Sci. Sport..

[6] Jonsson M., Hurtig-Wennlöf A., Ahlsson A., Vidlund M., Cao Y., Westerdahl E. (2019). In-hospital physiotherapy improves physical activity level after lung cancer surgery: A randomized controlled trial. Physiotherapy.

[7] McNarry M.A., Berg R.M., Shelley J., Hudson J., Saynor Z.L., Duckers J., Lewis K., Davies G.A., Mackintosh K.A. (2022). Inspiratory muscle training enhances recovery post-COVID-19: A randomised controlled trial. Eur. Respir. J..

[8] Wang J., Liu S., Li G., Xiao J. (2020). Exercise Regulates the Immune System. Adv. Exp. Med. Biol..

[9] Chastin S.F.M., Abaraogu U., Bourgois J.G., Dall P.M., Darnborough J., Duncan E., Dumortier J., Pavón D.J., McParland J., Roberts N.J. (2021). Effects of Regular Physical Activity on the Immune System, Vaccination and Risk of Community-Acquired Infectious Disease in the General Population: Systematic Review and Meta-Analysis. Sport. Med..

[10] Nieman D.C., Henson D.A., Austin M.D., Sha W. (2011). Upper respiratory tract infection is reduced in physically fit and active adults. Br. J. Sport. Med..

[11] Wong C.-M., Lai H.-K., Ou C.-Q., Ho S.-Y., Chan K.-P., Thach T.-Q., Yang L., Chau Y.-K., Lam T.-H., Hedley A.J. (2008). Is exercise protective against influenza-associated mortality?. PLoS ONE.

[12] Stockwell S., Trott M., Tully M., Shin J., Barnett Y., Butler L., McDermott D., Schuch F., Smith L. (2021). Changes in physical activity and sedentary behaviours from before to during the COVID-19 pandemic lockdown: A systematic review. BMJ Open Sport Exerc. Med..

[13] Pérez-Gisbert L., Torres-Sánchez I., Ortiz-Rubio A., Calvache-Mateo A., López-López L., Cabrera-Martos I., Valenza M.C. (2021). Effects of the COVID-19 Pandemic on Physical Activity in Chronic Diseases: A Systematic Review and Meta-Analysis. Int. J. Environ. Res. Public Health.

[14] Zaccagni L., Toselli S., Barbieri D. (2021). Physical Activity during COVID-19 Lockdown in Italy: A Systematic Review. Int. J. Environ. Res. Public Health.

[15] Mascherini G., Catelan D., Pellegrini-Giampietro D.E., Petri C., Scaletti C., Gulisano M. (2021). Changes in physical activity levels, eating habits and psychological well-being during the Italian COVID-19 pandemic lockdown: Impact of socio-demographic factors on the Florentine academic population. PLoS ONE.

[16] Sallis R., Young D.R., Tartof S.Y., Sallis J.F., Sall J., Li Q., Smith G.N., A Cohen D. (2021). Physical inactivity is associated with a higher risk for severe COVID-19 outcomes: A study in 48 440 adult patients. Br. J. Sport. Med..

[17] Lee S.W., Lee J., Moon S.Y., Jin H.Y., Yang J.M., Ogino S., Song M., Hong S.H., Ghayda R.A., Kronbichler A. (2022). Physical activity and the risk of SARS-CoV-2 infection, severe COVID-19 illness and COVID-19 related mortality in South Korea: A nationwide cohort study. Br. J. Sport. Med..

[18] Mohamed A.A., Alawna M. (2021). The effect of aerobic exercise on immune biomarkers and symptoms severity and progression in patients with COVID-19: A randomized control trial. J. Bodyw. Mov. Ther..

[19] Carfì A., Bernabei R., Landi F. (2020). Gemelli Against COVID-19 Post-Acute Care Study Group. Persistent Symptoms in Patients After Acute COVID-19. JAMA.

[20] Delbressine J., Machado F., Goërtz Y., Van Herck M., Meys R., Houben-Wilke S., Burtin C., Franssen F., Spies Y., Vijlbrief H. (2021). The Impact of Post-COVID-19 Syndrome on Self-Reported Physical Activity. Int. J. Environ. Res. Public Health.

[21] Asadi-Pooya A.A., Akbari A., Emami A., Lotfi M., Rostamihosseinkhani M., Nemati H., Barzegar Z., Kabiri M., Zeraatpisheh Z., Farjoud-Kouhanjani M. (2021). Risk Factors Associated with Long COVID Syndrome: A Retrospective Study. Iran. J. Med. Sci..

[22] Ahmed H., Patel K., Greenwood D., Halpin S., Lewthwaite P., Salawu A., Eyre L., Breen A., O’Connor R., Jones A. (2020). Long-term clinical outcomes in survivors of severe acute respiratory syndrome and Middle East respiratory syndrome coronavirus outbreaks after hospitalisation or ICU admission: A systematic review and meta-analysis. J. Rehabil. Med..

[23] Gemelli Against COVID-19 Post-Acute Care Study Group (2020). Post-COVID-19 global health strategies: The need for an interdisciplinary approach. Aging Clin. Exp. Res..

[24] World Health Organization (2020). Criteria for Releasing COVID-19 Patients from Isolation. https://www.who.int/news-room/commentaries/detail/criteria-for-releasing-covid-19-patients-from-isolation.

[25] Tajbakhsh A., Gheibi Hayat S.M., Taghizadeh H., Akbari A., Inabadi M., Savardashtaki A., Johnston T.P., Sahebkar A. (2021). COVID-19 and cardiac injury: Clinical manifestations, biomarkers, mechanisms, diagnosis, treatment, and follow up. Expert Rev. Anti. Infect. Ther..

[26] Wang F., Kream R.M., Stefano G.B. (2020). Long-Term Respiratory and Neurological Sequelae of COVID-19. Med. Sci. Monit..

[27] Aghagoli G., Gallo Marin B., Katchur N.J., Chaves-Sell F., Asaad W.F., Murphy S.A. (2021). Neurological Involvement in COVID-19 and Potential Mechanisms: A Review. Neurocrit. Care.

[28] Amin M. (2020). COVID-19 and the liver: Overview. Eur. J. Gastroenterol. Hepatol..

[29] Galluzzo V., Ciciarello F., Tosato M., Zazzara M.B., Pais C., Savera G., Calvani R., Picca A., Marzetti E., Landi F. (2022). Association between vitamin D status and physical performance in COVID-19 survivors: Results from the Gemelli against COVID-19 post-acute care project. Mech. Ageing Dev..

[30] U.S. Department of Health and Human Services (2018). Physical Activity Guidelines for Americans.

[31] Landi F., Calvani R., Martone A.M., Salini S., Zazzara M.B., Candeloro M., Coelho-Junior H.J., Tosato M., Picca A., Marzetti E. (2020). Normative values of muscle strength across ages in a “real world” population: Results from the longevity check-up 7+ project. J. Cachexia Sarcopenia Muscle.

[32] Briand J., Behal H., Chenivesse C., Wémeau-Stervinou L., Wallaert B. (2018). The 1-minute sit-to-stand test to detect exercise-induced oxygen desaturation in patients with interstitial lung disease. Ther. Adv. Respir. Dis..

[33] Zanini A., Aiello M., Cherubino F., Zampogna E., Chetta A., Azzola A., Spanevello A. (2015). The one repetition maximum test and the sit-to-stand test in the assessment of a specific pulmonary rehabilitation program on peripheral muscle strength in COPD patients. Int. J. Chronic Obstr. Pulm. Dis..

[34] Guyatt G.H., Sullivan M.J., Thompson P.J., Fallen E.L., Pugsley S.O., Taylor D.W., Berman L.B. (1985). The 6-minute walk: A new measure of exercise capacity in patients with chronic heart failure. Can. Med. Assoc. J..

[35] de Boer A.G., van Lanschot J.J., Stalmeier P.F., van Sandick J.W., Hulscher J.B., de Haes J.C., Sprangers M.A. (2004). Is a single-item visual analogue scale as valid, reliable and responsive as multi-item scales in measuring quality of life?. Qual. Life Res..

[36] Prete M., Luzzetti A., Augustin L.S.A., Porciello G., Montagnese C., Calabrese I., Ballarin G., Coluccia S., Patel L., Vitale S. (2021). Changes in Lifestyle and Dietary Habits during COVID-19 Lockdown in Italy: Results of an Online Survey. Nutrients.

[37] Notarte K.I., de Oliveira M.H.S., Peligro P.J., Velasco J.V., Macaranas I., Ver A.T., Pangilinan F.C., Pastrana A., Goldrich N., Kavteladze D. (2022). Age, Sex and Previous Comorbidities as Risk Factors Not Associated with SARS-CoV-2 Infection for Long COVID-19: A Systematic Review and Meta-Analysis. J. Clin. Med..

[38] Alkodaymi M.S., Omrani O.A., Fawzy N.A., Shaar B.A., Almamlouk R., Riaz M., Obeidat M., Obeidat Y., Gerberi D., Taha R.M. (2022). Prevalence of post-acute COVID-19 syndrome symptoms at different follow-up periods: A systematic review and meta-analysis. Clin. Microbiol. Infect..

[39] Sandler C.X., Wyller V.B.B., Moss-Morris R., Buchwald D., Crawley E., Hautvast J., Katz B.Z., Knoop H., Little P., Taylor R. (2021). Long COVID and Post-infective Fatigue Syndrome: A Review. Open Forum Infect. Dis..

[40] Stengel A., Malek N., Zipfel S., Goepel S. (2022). Long Haulers—What Is the Evidence for Post-COVID Fatigue?. Front. Psychiatry.

[41] Tosato M., Calvani R., Picca A., Ciciarello F., Galluzzo V., Coelho-Júnior H.J., Di Giorgio A., Di Mario C., Gervasoni J., Gremese E. (2022). Effects of l-Arginine Plus Vitamin C Supplementation on Physical Performance, Endothelial Function, and Persistent Fatigue in Adults with Long COVID: A Single-Blind Randomized Controlled Trial. Nutrients.

[42] Galluzzo V., Zazzara M.B., Ciciarello F., Savera G., Pais C., Calvani R., Picca A., Marzetti E., Landi F., Tosato M. (2022). Fatigue in Covid-19 survivors: The potential impact of a nutritional supplement on muscle strength and function. Clin. Nutr. ESPEN.

[43] Landi F., Martone A.M., Ciciarello F., Galluzzo V., Savera G., Calvani R., Picca A., Marzetti E., Tosato M., On behalf of Gemelli Against COVID-19 Post-Acute Care Team (2022). Effects of a New Multicomponent Nutritional Supplement on Muscle Mass and Physical Performance in Adult and Old Patients Recovered from COVID-19: A Pilot Observational Case–Control Study. Nutrients.

[44] Wright J., Astill S.L., Sivan M. (2022). The Relationship between Physical Activity and Long COVID: A Cross-Sectional Study. Int. J. Environ. Res. Public Health.

[45] Lombardo M.D.M., Foppiani A., Peretti G.M., Mangiavini L., Battezzati A., Bertoli S., Boneschi F.M., Zuccotti G.V. (2021). Long-Term Coronavirus Disease 2019 Complications in Inpatients and Outpatients: A One-Year Follow-up Cohort Study. Open Forum Infect. Dis..

[46] Bhutani S., vanDellen M.R., Cooper J.A. (2021). Longitudinal Weight Gain and Related Risk Behaviors during the COVID-19 Pandemic in Adults in the US. Nutrients.

[47] Sardeli A.V., Tomeleri C.M., Cyrino E.S., Fernhall B., Cavaglieri C.R., Chacon-Mikahil M.P.T. (2018). Effect of resistance training on inflammatory markers of older adults: A meta-analysis. Exp. Gerontol..

[48] Tir A.M.D., Labor M., Plavec D. (2017). The effects of physical activity on chronic subclinical systemic inflammation. Arch. Ind. Hyg. Toxicol..

[49] Gleeson M., Bishop N.C., Stensel D.J., Lindley M.R., Mastana S.S., Nimmo M.A. (2011). The anti-inflammatory effects of exercise: Mechanisms and implications for the prevention and treatment of disease. Nat. Rev. Immunol..

[50] Simpson R.J., Kunz H., Agha N., Graff R. (2015). Exercise and the Regulation of Immune Functions. Prog. Mol. Biol. Transl. Sci..

[51] Schipper H.S., Prakken B., Kalkhoven E., Boes M. (2012). Adipose tissue-resident immune cells: Key players in immunometabolism. Trends Endocrinol. Metab..

[52] Hansen A.L., Ambroziak G., Thornton D.M., Mundt J.C., Kahn R.E., Dahl L., Waage L., Kattenbraker D., Grung B. (2021). Vitamin D Status and Physical Activity during Wintertime in Forensic Inpatients-A Randomized Clinical Trial. Nutrients.

[53] Levis S., Gómez-Marín O. (2017). Vitamin D and Physical Function in Sedentary Older Men. J. Am. Geriatr. Soc..

[54] Bislev L.S., Langagergaard Rødbro L., Rolighed L., Sikjaer T., Rejnmark L. (2018). Effects of Vitamin D3 Supplementation on Muscle Strength, Mass, and Physical Performance in Women with Vitamin D Insufficiency: A Randomized Placebo-Controlled Trial. Calcif. Tissue Int..

[55] Polly P., Tan T.C. (2014). The role of vitamin D in skeletal and cardiac muscle function. Front. Physiol..

[56] Samefors M., Tengblad A., Östgren C.J. (2020). Sunlight Exposure and Vitamin D Levels in Older People—An Intervention Study in Swedish Nursing Homes. J. Nutr. Health Aging.

[57] Townsend L., Dyer A.H., McCluskey P., O’Brien K., Dowds J., Laird E., Bannan C., Bourke N.M., Cheallaigh C.N., Byrne D.G. (2021). Investigating the Relationship between Vitamin D and Persistent Symptoms Following SARS-CoV-2 Infection. Nutrients.

[58] Bohannon R.W. (2017). Crouch R. Minimal clinically important difference for change in 6-minute walk test distance of adults with pathology: A systematic review. J. Eval. Clin. Pract..

[59] Falck R.S., Davis J.C., Best J.R., Crockett R.A., Liu-Ambrose T. (2019). Impact of exercise training on physical and cognitive function among older adults: A systematic review and meta-analysis. Neurobiol. Aging.

[60] Paneroni M., Simonelli C., Saleri M., Bertacchini L., Venturelli M., Troosters T., Ambrosino N., Vitacca M. (2021). Muscle Strength and Physical Performance in Patients Without Previous Disabilities Recovering From COVID-19 Pneumonia. Am. J. Phys. Med. Rehabil..

[61] Martone A.M., Tosato M., Ciciarello F., Galluzzo V., Zazzara M.B., Pais C., Savera G., Calvani R., Marzetti E., Robles M.C. (2022). Sarcopenia as potential biological substrate of long COVID-19 syndrome: Prevalence, clinical features, and risk factors. J. Cachexia Sarcopenia Muscle.

[62] Awick E.A., Ehlers D.K., Aguiñaga S., Daugherty A.M., Kramer A.F., McAuley E. (2017). Effects of a randomized exercise trial on physical activity, psychological distress and quality of life in older adults. Gen. Hosp. Psychiatry.

[63] Groessl E.J., Kaplan R.M., Rejeski W.J., Katula J.A., Glynn N.W., King A.C., Anton S.D., Walkup M., Lu C.-J., Reid K. (2019). Physical Activity and Performance Impact Long-term Quality of Life in Older Adults at Risk for Major Mobility Disability. Am. J. Prev. Med..

[64] Tosato M., Ciciarello F., Zazzara M.B., Janiri D., Pais C., Cacciatore S., Montenero R., Leone M.S., Chisci E., Picca A. (2022). Lifestyle Changes and Psychological Well-Being in Older Adults During COVID-19 Pandemic. Clin. Geriatr. Med..

[65] Fernández-de-Las-Peñas C., Notarte K.I., Peligro P.J., Velasco J.V., Ocampo M.J., Henry B.M., Arendt-Nielsen L., Torres-Macho J., Plaza-Manzano G. (2022). Long-COVID Symptoms in Individuals Infected with Different SARS-CoV-2 Variants of Concern: A Systematic Review of the Literature. Viruses.

